# Application of Acellular Tissue Matrix for Enhancement of Weak Abdominal Wall in Animal Model

**DOI:** 10.1155/2020/3475289

**Published:** 2020-03-11

**Authors:** Minggang Wang, Shuo Yang, Zhen Cao, Sanyuan Hu

**Affiliations:** ^1^Department of General Surgery, Shandong Provincial Qianfoshan Hospital, Shandong University, Jinan, Shandong 250014, China; ^2^Department of Hernia and Abdominal Wall Surgery, Beijing Chaoyang Hospital, Capital Medical University, Beijing 100043, China

## Abstract

**Background:**

Abdominal wall weakness occurs when the strength of muscle decreases due to physiological reason or iatrogenic injury. However, the treatment of this disease is complicated.

**Aim:**

To study the therapeutic effect of acellular tissue matrix (ACTM), compared with the polypropylene mesh.

**Methods:**

An abdominal wall weakness model was established in rabbits through motor nerves cutting. The polypropylene mesh and ACTM were implanted in the left and right abdomen sides, respectively. Mechanical testing of abdominal wall muscle and histology and scanning electron microscopy (SEM) evaluation of abdominal tissue explants were performed.

**Results:**

In animal model establishment, the abdominal length of healthy and weakened abdominal wall was 17.0 ± 0.7 cm and 19.0 ± 1.2 cm, respectively (*P*=0.022), and the weak abdominal wall group showed a significant decrease of 1.116 ± 0.221 MPa in tensile stress (*P*=0.022), and the weak abdominal wall group showed a significant decrease of 1.116 ± 0.221 MPa in tensile stress (*P*=0.022), and the weak abdominal wall group showed a significant decrease of 1.116 ± 0.221 MPa in tensile stress (*P*=0.022), and the weak abdominal wall group showed a significant decrease of 1.116 ± 0.221 MPa in tensile stress (*P*=0.022), and the weak abdominal wall group showed a significant decrease of 1.116 ± 0.221 MPa in tensile stress (*P*=0.022), and the weak abdominal wall group showed a significant decrease of 1.116 ± 0.221 MPa in tensile stress (*P*=0.022), and the weak abdominal wall group showed a significant decrease of 1.116 ± 0.221 MPa in tensile stress (

**Conclusion:**

The abdominal wall weakness model in rabbits was successfully established. ACTM is a promising biological material to be possibly further applied in clinical surgery in patients with abdominal wall weakness.

## 1. Introduction

The abdominal wall is made up of muscle and attaching tissues, which provide strength to hold the contents in the abdominal cavity [1]. Hernias are pretty common and debilitating, with a high rate of application for biomaterials engineering [2]. Abdominal wall hernia, a disease when a defect occurs in the abdominal wall allowing for the abdominal organs being pushed into the outside, is one of the most often performed surgical procedures worldwide [1, 3]. Different from abdominal wall hernia, abdominal wall weakness occurs when the strength of muscle decreases due to physiological reason or iatrogenic injury. With the increase of lumbar surgery, especially retroperitoneal surgery after urology, the incidence of nerve injury that caused a weak abdominal wall is increasing. The main cause is the denervation of the abdominal wall muscle, resulting in the lack of strength of the muscular layer to resist the increasing abdominal pressure. As a consequence, local bulging appears with progressive aggravation, leading to lumbar eventration. In severe cases, there may be a prolapse of the abdominal organs, causing great pain to the patients.

Mesh material is commonly used in abdominal wall hernia and incisional hernia repair, and the implantation of mesh has been shown to reduce hernia recurrence significantly [4]. Although the introduction of mesh materials has improved outcomes of surgery, there are still problems including pain, infection, and recurrence that trouble patients [2]. From clinical practice and experience, the treatment for abdominal wall weakness is even more difficult than that for abdominal wall hernia, because the place of mesh is hard to determine without clear hernia ring existing. Moreover, the mesh used in abdominal hernia repair is less suitable for the weak abdominal wall treatment. Based on the scar repair to tighten surrounding tissues, the classical polypropylene mesh results in the decrease of local abdominal wall compliance after healing and brings strong discomfort when patients move the waist [5]. Besides, the increasing risk of infection from effusion stimulated by exogenous polypropylene mesh and unsatisfied therapeutic improvement of abdominal muscle strength after polypropylene mesh implantation are in urgent need to be solved.

The acellular tissue matrix is a newly developed tissue repairing material in recent years, which mainly uses the same or different kinds of skin, pericardium tissue, intestinal submucosal tissue, or other collagen matrices after decellularization and removal of immunogenicity through different methods [6]. The three-dimensional framework of the extracellular matrix of the tissue is completely retained and can stimulate and induce the own fibroblasts of the body to grow, secrete collagen, and finally complete the repair of tissue defects [6]. Meanwhile, the biomaterial scaffold itself can be gradually degraded and absorbed and utilized by the body, since the main component of the acellular matrix repair material is protein including collagen fibers, glycoproteins, and mucins [7]. The mechanism of acellular tissue matrix repair is that the implanted biological materials gradually degrade and are replaced by host tissue with the two processes almost in synchronization, unlike the mechanism of traditional nonabsorbent synthetic polymer material patch repair relying on the body inflammation to stimulate the formation of continuously enhanced fibrotic scar tissue [5, 8]. More importantly, the acellular tissue matrix attracts and induces stem cells to secrete the extracellular matrix to replace the gradually degraded biomaterial. Therefore, the biological acellular tissue matrix has the advantages of self-degradability, good biocompatibility, and light tissue adhesion without excessive scar formation and long-term chronic inflammation [9–11].

Herein, we firstly established a weak abdominal wall model in rabbits by transverse injury of motor nerves leading to the denervation of the abdominal wall muscle. After 12 weeks, acellular tissue matrix (ACTM) and polypropylene mesh as control were implanted into the already established weak abdominal wall. After 24 and 48 weeks, respectively, abdominal muscle tissue was taken out for biomechanical, scanning electron microscope (SEM), or histology evaluation.

## 2. Materials and Methods

### 2.1. Establishment of Weak Abdominal Wall Model in Rabbits

**Figure 1:**
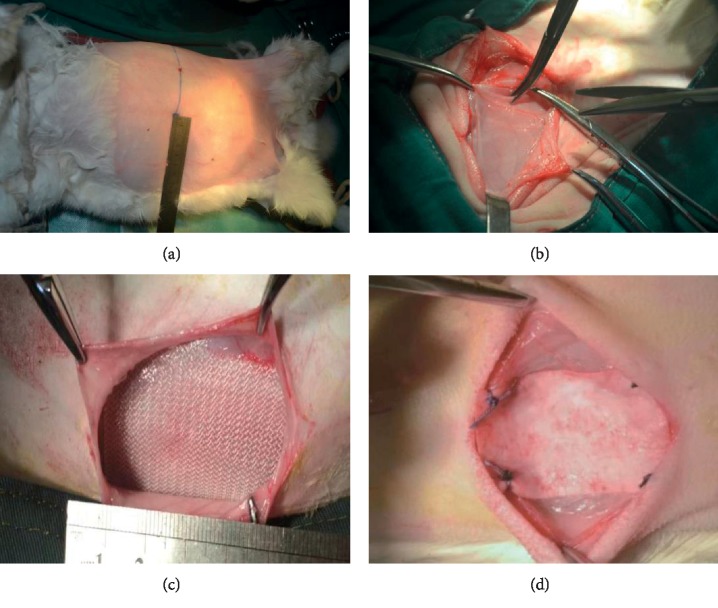
Representative images of the surgical procedure showing the experimental position to establish weak abdominal wall animal model and measurement of abdominal length (a), the excision of abdominal wall motor nerve in surgery (b), and implantation of mesh (c), (d). Red circle indicates the surgery area under measuring ruler. Red arrow points at the motor nerve.

Twenty healthy 1-year-old New Zealand white rabbits weighing 3-4 kg (provided by the Animal Experimental Center of the National Engineering Laboratory of Guanhao Biotechnology Co., Ltd.) were used to establish the weak abdominal wall model. This study was approved and conducted in accordance with the guidelines of the Animal Ethics Committee of Capital Medical University. Both sides of the abdominal wall were studied as a control and experimental group. The motor nerves of the abdominal wall were cut for the establishment of this model. To look for motor nerves, one side of the abdominal wall was separated along the direction of the external oblique muscle. After finding them, we cut the adjacent three nerves and sutured the skin subcutaneously. Meanwhile, the other side of the abdominal wall was operated as described above without cutting the nerves. During the period of observation, animals were well treated and maintained. After 12 weeks, the length of the bilateral abdominal wall (from the posterior axillary line to the anterior median line) was recorded. Then, both sides of the abdominal muscle (about 8 × 8 cm) were taken out and prepared for mechanical testing including tensile stress and strain. The representative images of the surgical procedure were shown in Figure 1.

### 2.2. Preparation and Source of Implanted Materials

The commercially available monofilament polypropylene woven mesh (Becton, Dickinson and Company, NJ, USA) was used as classical implanted material for comparison. The acellular tissue matrix (Type B thoracic surgery repair film; Guanhao Biotechnology Co, Ltd, Guangzhou, China) was used as novel implanted material. Both materials were prepared in advance to be in the suitable size.

### 2.3. Preoperative Preparation

The animals were fasted and water-deprived for 8 hours before surgery. The shaved area of skin was up to 2 cm below the armpit, down to the level of the pubic tubercle, bilateral to the posterior axillary line. The animal was placed in a supine position with the lower limbs fixed.

### 2.4. Material Implantation

Injection of 3% pentobarbital sodium into the vein was used to anesthetize the animals. Firstly, the skin within the surgery area was incised. After proper exposure, polypropylene mesh and ACTM were implanted in the posterior muscle space of each side. Then, the implanted materials were sutured and fixed, and the defect area was repaired.

### 2.5. Postoperative Management

To prevent infection, each animal was orally treated with penicillin for 3 days after surgery. During this period, the body temperature and the local wound condition were monitored, and postoperative diet control was taken.

### 2.6. Biomechanical Test

The biomechanical test followed Lai et al. and others' method [12–16]. Briefly, the biomechanical properties of the subcutaneous materials-implanted tissue samples were evaluated within 2 h while stored in PBS at 4°C. A separation distance of more than 10 mm was used in fixing the samples with clamps and a 50 mm min^−1^ uniaxial tensile force was exerted. The sample width is 4.0 ± 0.1 mm. The electronic material testing system (Instron, USA; #3343) was used for mechanical testing. First, we cut the material into a strip of a certain width and measured the thickness of the material. Then, we input the thickness and width values into the test software. Finally, we clamped the material on the upper and lower clamps (with a distance of 0.5 mm) of the mechanical testing machine and stretched it at a stable speed. When the sample was stretched to fracture, the test ended. Tensile stress and tensile strain were then obtained and recorded.

### 2.7. Histologic Analysis

To determine the morphological changes of cells around the implanted materials, hematoxylin and eosin (H&E) staining was carried out. The procedure was followed as previously reported [17]. At 24 weeks after implantation, the surrounding tissues of the surgery area were collected. Firstly, samples were fixed in 10% buffered formalin at 25°C for 48 h. After embedding in paraffin, they were cut into slices of 5 *μ*m. Finally, the slides were stained with hematoxylin and eosin, viewed using a microscope (Olympus, USA), and recorded by image processing software.

### 2.8. SEM Evaluation

The procedure was followed as previously reported [10, 12, 18]. 24 weeks and 48 weeks after implantation, the subcutaneous materials-implanted tissue samples were taken out for SEM evaluation. To prepare the samples, implanted materials were separated, rinsed twice with PBS, and fixed with 3% glutaraldehyde solution at 4°C for 2 h. Then, the samples were washed with PBS twice to remove the glutaraldehyde solution. For dehydration, the acetone/isoamyl acetate (1 : 1) was added. After 10 min, 100% isoamyl acetate was added and kept for 30 min. After being soaked in 50%, 70%, 80%, 90%, 95%, and 100% acetonitrile solutions successively and dried in a vacuum oven, the samples were ready for observation. Finally, the samples were sputter-coated with a thin layer of gold and observed by a scanning electron microscopy (SEM) with a magnification of 200x and 1000x at a voltage of 20 kV.

### 2.9. Statistical Analysis

**Figure 2:**
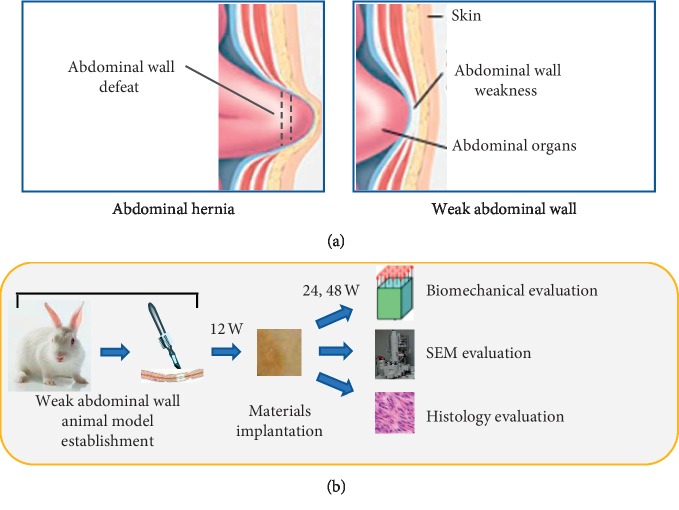
(a) Schematic illustration of the difference between abdominal hernia and weak abdominal wall. (b) Schematic illustration of the animal model establishment, material application, and evaluations.

SPSS software version 20 (IBM Corp., New York, NY, USA) was used for statistical analysis. The Kolmogorov-Smirnov and Shapiro-Wilk tests were performed to test the normality of biomechanical data. A paired *t*-test was used to analyze the data of weak abdominal wall animal model establishment and rabbits implanted with polypropylene and ACTM after 24 and 48 weeks. Levene's test for the equation of variance (ANOVA) and independent samples *t*-test were used to analyze the data of biomechanical change in rabbits implanted with polypropylene and ACTM from 24 weeks to 48 weeks. All tests were 2-sided. *P* < 0.05 was considered to be statistically significant. The difference between abdominal hernia and weak abdominal wall was shown in Figure 2(a), while the experimental procedure was shown in Figure 2(b).

## 3. Results

### 3.1. Procedure Results

In the establishment of the weak abdominal wall model, two rabbits died due to an anesthesia accident and one rabbit died due to postoperative infection, with three supplementary rabbits afterwards. No bleeding happened and all rabbits survived postoperatively.

In the experiments of materials implantation, two rabbits in the polypropylene mesh group and two rabbits in the ACTM group died due to an anesthesia accident, with four supplementary rabbits afterwards. Four rabbits in the polypropylene mesh group and one rabbit in the ACTM group got infected after materials implantation and recovered after debridement and suture. Polypropylene mesh and ACTM materials were successfully implanted with a rate of 100% (30/30 for polypropylene mesh implantation in one side, 30/30 for ACTM implantation on the other side). All rabbits survived after surgery.

### 3.2. Establishment of Weak Abdominal Wall Model and Mesh Implantation

Abdominal length and biomechanical properties including tensile stress and tensile strain were measured. The abdominal length of the healthy and weakened abdominal wall was 17.0 ± 0.7 cm and 19.0 ± 1.2 cm, respectively (*P*=0.022), indicating an average increase of 2 cm in the weak abdominal wall animal model. Furthermore, the mesh was implanted.

**Figure 3:**
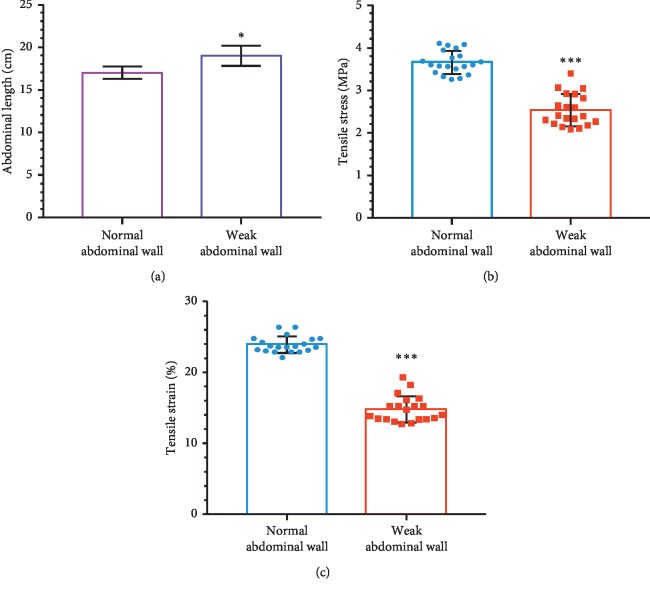
Abdominal length (a) and biomechanical evaluation including tensile stress (b) and tensile strain (c) of weak abdominal wall animal model establishment. Data are shown as mean ± SD. ^*∗*^*P* < 0.05. ^*∗∗∗*^*P* < 0.001.

By analyzing the biomechanical data (Table 1), the differences in tensile stress and tensile strain between normal and weak abdominal wall were in normality (Table 2). Paired *t*-test was further performed and the weak abdominal wall group showed a significant decrease of 1.116 ± 0.221 MPa in tensile stress (*P* < 0.001) and a significant decrease of 9.126 ± 2.073% in tensile strain (*P* < 0.001). As shown in Figure 3, both abdominal length and biomechanical properties confirmed the successful establishment of the weak abdominal wall model.

### 3.3. Mechanical Testing

**Figure 4:**
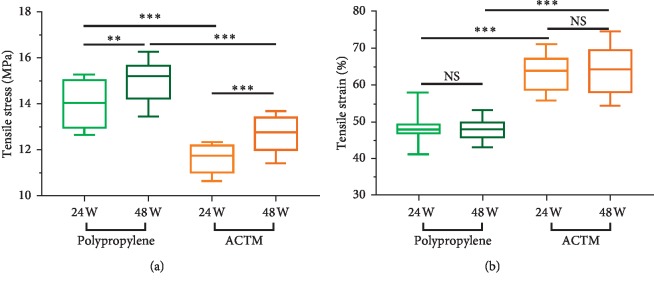
Biomechanical evaluation including tensile stress (a) and tensile strain (b) of rabbits implanted with monofilament polypropylene woven mesh (polypropylene) and acellular tissue matrix (ACTM) after 24 and 48 weeks. Data are shown as mean ± SD. ^*∗∗*^*P* < 0.01. ^*∗∗∗*^*P* < 0.001.

To investigate the biomechanical properties of muscle tissues surrounding implanted materials, tensile stress and tensile strain were measured and compared. Biomechanical data of rabbits implanted with polypropylene and ACTM after 24 weeks and 48 weeks was shown in Tables 3 and 4, respectively. After confirming that the differences of tensile stress and tensile strain between polypropylene group and ACTM group were in normality, pair *t*-test was further carried out (Tables 5 and 6). Meanwhile, the changes in tensile stress and tensile strain of each group from 24 weeks to 48 weeks were analyzed as well (Table 7). As shown in Figure 4(a), compared with the polypropylene group, average tensile stress decreased to 2.409 ± 0.806 MPa after 24 weeks and 2.319 ± 0.486 MPa after 48 weeks in ACTM group (*P*=1.482*E* − 08 and *P*=3.093*E* − 11, respectively). Notably, an average increase of 1.01 ± 0.321 MPa and 1.1 ± 0.244 MPa was observed in the polypropylene group (*P*=0.004) and the ACTM group (*P* < 0.001), respectively, from 24 weeks to 48 weeks. As shown in Figure 4(b), compared with the polypropylene group, the average tensile strain increased to 15.259% after 24 weeks (*P*=2.992*E* − 07) and 15.845% after 48 weeks (*P*=2.900*E* − 07) remarkably. But no statistical significance was found in the change of each group from 24 weeks to 48 weeks.

### 3.4. Histology Evaluation

**Figure 5:**
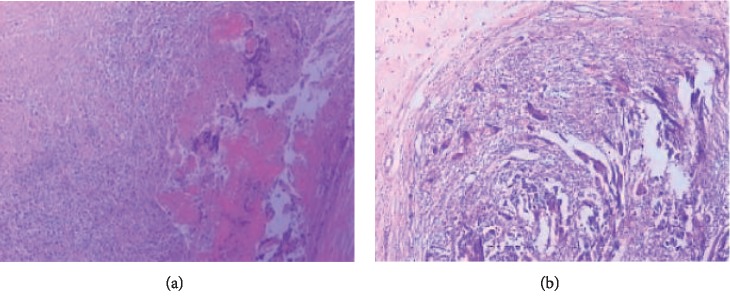
Histology evaluation by H&E staining of tissues surrounding implantation materials from representative animals after 12 weeks (at 100x original magnification). The polypropylene group (a) showed an inflammatory reaction around implanted mesh with lymphocytes and plasmocytes more than 50/HPF. The ACTM group (b) showed improved biological degradation, no rejection reaction with lymphocytes and plasmocytes less than 25/HPF, and granulation tissue fibrosis inside.

Representative images of H&E stained slides of abdominal tissue explants are shown in Figure 5. After 24 weeks, slight inflammatory reactions were found around polypropylene mesh and more than 50 immune cells including lymphocytes and plasmocytes were observed per high-power field. However, the ACTM group showed almost no inflammatory reaction with inflammatory cells less than 25 per high-power field and granulation tissue inside, indicating an improved biological degradation. Also, better development of blood capillary was encouraged. Moreover, the tissue structure was closer to the normal and physiological condition with narrow tissue gap and homogeneous staining around implanted ACTM unlike polypropylene group, suggesting outstanding biocompatibility.

### 3.5. SEM Evaluation

**Figure 6:**
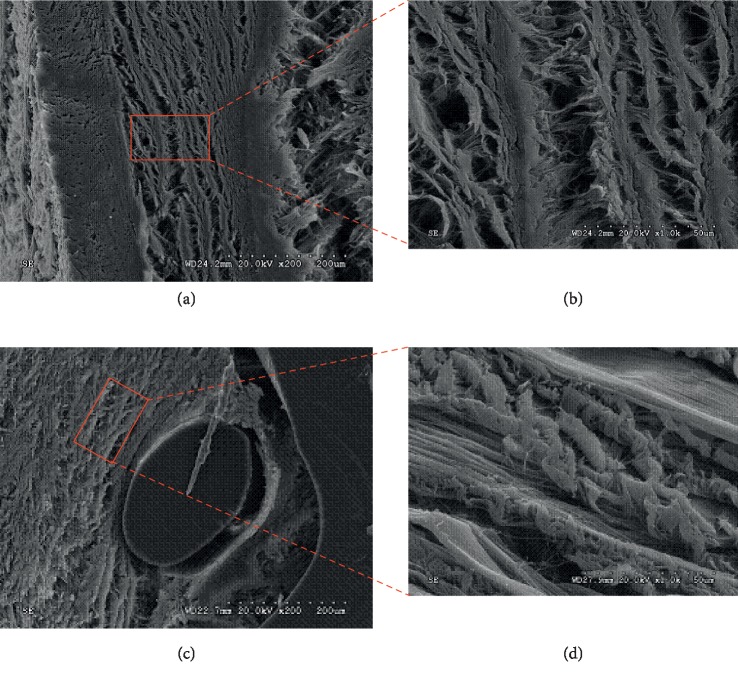
SEM images of ACTM implantation after 12 weeks with magnification of 200x (a) and 1000x (b) and 24 weeks with magnification of 200x (c) and 1000x (d). Red square indicates the specific area of magnification. After 12 weeks, the fiber arrangement and orientation were relatively neat and normal (a), (b). After 24 weeks, the outer edge showed a certain degree of degradation with the arrangement of the collagen fibers less neatly (c), (d).

As already shown by biomechanical and histology evaluation, ACTM exhibited a better therapeutic effect. Thus, SEM evaluation was finally performed to observe the microstructure of abdominal tissue explants in shape. Two-time points including 24 weeks and 48 weeks were chosen (Figure 6). The fibrillar shape of ACTM (middle part in Figure 6(a) and magnification in Figure 6(b); left part in Figure 6(c) and magnification in Figure 6(d)) could be clearly observed. After 24 weeks, the fiber arrangement and orientation were relatively neat and normal. Furthermore, it can be seen that the cells adhered to the ACTM and the fibers were intertwined. No obvious structural changes were found. After 48 weeks, the outer edge showed a certain degree of degradation with surrounding tissue growing into it.

Compared with 24 weeks ago, the collagen fibers were arranged a little bit less neatly. It can be seen that the structure of implanted ACTM changed slightly, and the bundles were still closely connected.

## 4. Discussion

Acellular tissue matrix has been widely studied and applied in different areas. According to the source, acellular tissue matrix could be classified into the acellular dermal matrix, acellular adipose matrix, acellular bone matrix, and so forth [19–21]. Based on the specific property, each kind of acellular tissue matrix has been applied in different diseases. For instance, articles published in The Lancet Oncology have involved cellular dermal matrix in breast construction and suggested its promising value in a clinical trial [22, 23]. A recent study showed the biological function of the decellularized extracellular matrix in renal tissue regeneration [24]. Acellular tissue matrix also has wide and practical applications in plastic, especially in the repair and reconstruction of bone, urethral, and vagina, due to the share of similar biological properties [7, 25, 26].

Given that most acellular tissue matrices used in abdominal surgery are for hernia repair and abdominal wall defects (especially in pediatrics) [27–29], our study is in great novelty to study the application of acellular tissue matrix in the treatment of abdominal wall weakness. As previously introduced, it is even harder to treat abdominal wall weakness than abdominal wall hernia because of the different pathogenesis. Nevertheless, not much attention has been paid to this disease. Smith et al. studied abdominal wall weakness caused by prune belly syndrome in children [30]. Reda et al. reported a case with paresthesia and weakness of the abdominal wall in old women [31]. Jangö et al. found that tissue-engineering with muscle fiber fragments could improve the strength of a weak abdominal wall [17]. Considering the abdominal wall weakness unsolved in clinical practice and troubling patients, a weak abdominal wall model in rabbits is first established. In body size, rabbits are more appropriate for motor nerves cutting, materials implantation, and other operations in the abdominal wall than other animals such as pigs and rats [17, 32]. The significant increase in abdominal length, tensile stress, and tensile strain together indicated that the strength of the abdominal muscle was obviously weakened, thus leading to the weak abdominal wall. The successful establishment of our weak abdominal wall animal model provided a solid foundation for further in vivo study.

The results of mechanical testing suggested a better effect of the ACTM in the improvement of passive mechanical property over the traditional polypropylene mesh as the tensile strain was much higher after 24 and 48 weeks. The tensile stress increased from 24 weeks to 48 weeks in both groups, indicating a repairing process of the abdominal wall with the help of polypropylene mesh and ACTM. Although the strength of abdominal wall muscle gradually recovered in both groups, the ACTM group seemed to have a better recovery as the average tensile stress increased more during the period. What's more, the tensile stress in the ACTM group was much lower than that in the polypropylene group with a decrease of 17.18% after 24 weeks and 15.25% after 48 weeks. Meanwhile, it is known that the use of ACTM implants does not allow the recovery of muscle contraction ability since the abdominal wall weakness is a muscle impairment resulting from nerve injury. Of note, muscle contraction as a biomechanical property plays a role in the healthy abdominal wall [33], and the application of biological prostheses may bring possible advantages in recovering muscular tissue [34].

It is known that the mesh can help the abdominal wall to withstand exerted physiological pressure; however, the large tensile strain was reported to possibly induce a modification and change the mechanical behavior [35]. The stiffening behavior caused by large tensile strain may abate the abdominal wall elasticity and compliance, leading to much discomfort and pain for patients with abdominal wall weakness [35]. Therefore, compared to traditional polypropylene mesh, novel biological ACTM seemed to be more effective in the treatment of abdominal wall weakness while keeping more abdominal wall compliance. Nevertheless, too low membrane stiffness of the implanted materials is not able to provide enough support, so a balance in the proper tensile stress is necessary [36]. Of note, anisotropic of the abdominal wall is also a factor that needs to be paid attention to. As reported previously, significant differences in stiffness appeared between the craniocaudal axis and the lateral axis [37, 38].

Another problem troubling surgeons is the significant shrink after the implantation of materials. In rabbit models, Konerding et al. found that the shrinkage of implanted polypropylene mesh was about 1%–5% after 3 months [39]. Similarly, Novitsky et al. found a polypropylene mesh shrinkage of 5%–10% after 1 year [40]. According to the wound repair process, mesh shrinkage is different in diverse materials [41]. Biological ACTM may possibly reduce the shrinkage on the basis of its repairing mechanism different from polypropylene mesh. The polypropylene mesh is nonabsorbent; thus, the repair relies on the contraction of scar tissue [5]. The ACTM degrades while tissue grows; thus, the repair makes the use of tissue regeneration [41]. From this perspective, ACTM could bring patients with less comfort and pain. Furthermore, the interaction between implanted materials and tissues should be considered as well. The exogenous materials could activate a series of host reactions. In this complex process, a formation of granuloma aiming to isolate the implanted materials may occur, which influences the normal repair and decreases the therapeutic effect. To some extent, the ACTM shares some common characteristics with body tissue in components and structures, contributing to incredibly excellent biocompatibility [6].

## 5. Conclusion

This study firstly established an abdominal wall weakness model in rabbits through motor nerves cutting. By comparison of the implanted polypropylene mesh and ACTM, it was shown that ACTM was more effective in the treatment of abdominal wall weakness while keeping more abdominal wall compliance. Therefore, ACTM is a promising biological material to be possibly further applied in clinical surgery in patients with abdominal wall weakness.

## Figures and Tables

**Table 1:** Biomechanical data of weak abdominal wall animal model establishment.

	Tensile stress (MPa)		Tensile strain (%)	
	Normal	Weak	Normal	Weak
	3.26	2.12	22.98	19.40
	4.05	3.40	24.81	18.25
	4.11	2.93	25.30	17.10
	4.08	3.08	26.42	16.21
	3.95	3.05	22.05	15.09
	3.56	2.65	23.01	13.98
	3.28	2.22	23.24	14.79
	3.37	2.32	22.88	15.22
	3.68	2.41	24.07	16.24
	3.59	2.40	23.71	15.22
	3.56	2.36	23.50	13.92
	4.00	2.92	24.80	15.21
	3.77	2.82	24.22	13.3
	3.81	2.61	23.81	12.66
	3.68	2.16	24.70	12.82
	3.62	2.28	23.50	12.96
	3.42	2.61	26.40	13.44
	3.33	2.34	23.40	13.59
	3.65	2.19	22.90	13.42
	3.51	2.10	23.15	13.51
Mean	**3.664**	**2.549**	**23.943**	**14.817**
SD	**0.269**	**0.374**	**1.162**	**1.856**
SE	0.060	0.084	0.260	0.415

**Table 2:** Normality test and paired *t*-test analysis of weak abdominal wall animal model establishment.

Pair	Kolmogorov-Smirnov			Shapiro-Wilk			Mean	SD	SE	95% CI	*t*	*P* value
	Statistic	df	Sig.	Statistic	df	Sig.						
Tensile stress (MPa): normal-weak	0.101	20	0.200	**0.987**	20	**0.993**	**1.116**	**0.221**	0.050	1.012–1.219	22.528	**3.621E** **−** **15**
Tensile strain (%): normal-weak	0.118	20	0.200	**0.964**	20	**0.632**	**9.126**	**2.073**	0.464	8.156–10.096	19.688	**4.236E** **−** **14**

**Table 3:** Biomechanical data of rabbits implanted with polypropylene and ACTM after 24 weeks.

	Tensile stress (MPa)		Tensile strain (%)	
	Polypropylene	ACTM	Polypropylene	ACTM
	14.05	11.22	43.32	67.22
	12.65	10.65	50.25	60.34
	12.88	11.05	46.98	66.22
	15.02	12.08	47.44	66.56
	14.76	12.21	49.06	59.02
	14.00	10.98	48.10	58.84
	13.52	11.14	50.80	70.05
	15.14	12.32	47.96	71.14
	15.04	12.20	48.23	68.43
	12.98	12.34	48.21	65.23
	12.90	11.76	41.22	55.92
	13.45	11.91	47.08	57.28
	14.22	11.02	49.19	59.11
	14.43	11.30	58.00	58.48
	15.28	12.01	43.09	63.98
Mean	**14.021**	**11.613**	**47.929**	**63.188**
SD	**0.915**	**0.579**	**3.870**	**5.002**
SE	0.236	0.149	0.999	1.292

**Table 4:** Biomechanical data of rabbits implanted with polypropylene and ACTM after 48 weeks.

	Tensile stress (MPa)		Tensile strain (%)	
	Polypropylene	ACTM	Polypropylene	ACTM
	13.44	11.45	47.49	57.22
	14.02	11.41	48.27	59.92
	13.95	11.89	45.49	64.39
	15.20	12.54	45.38	66.33
	14.24	12.03	48.05	58.32
	14.55	13.03	51.23	55.35
	15.48	12.87	53.23	74.52
	14.92	12.65	50.17	73.26
	15.65	13.13	49.91	67.12
	15.92	13.68	47.05	59.44
	16.24	13.52	48.66	69.43
	14.81	13.66	47.42	61.22
	15.54	12.57	45.86	70.04
	15.39	12.79	43.21	67.33
	16.06	13.41	49.18	54.39
Mean	**15.027**	**12.709**	**48.040**	**63.885**
SD	**0.843**	**0.745**	**2.538**	**6.432**
SE	0.218	0.192	0.655	1.661

**Table 5:** Normality test and paired *t*-test analysis of rabbits implanted with polypropylene and ACTM after 24 weeks.

Pair	Kolmogorov-Smirnov			Shapiro-Wilk			Mean	SD	SE	95% CI	*t*	*P* value
	Statistic	df	Sig.	Statistic	df	Sig.						
Tensile stress (MPa): Polypropylene-ACTM	0.139	15	0.200	**0.921**	15	**0.2**	**2.409**	**0.806**	0.208	1.962–2.855	11.576	**1.482E**−**08**
Tensile strain (%): Polypropylene-ACTM	0.190	15	0.148	**0.913**	15	**0.15**	**−15.259**	**6.499**	1.678	**−**18.859–11.66	−9.093	**2.992E**−**07**

**Table 6:** Normality test and paired *t*-test analysis of rabbits implanted with polypropylene and ACTM after 48 weeks.

Pair	Kolmogorov-Smirnov			Shapiro-Wilk			Mean	SD	SE	95% CI	*t*	*P* Value
	Statistic	df	Sig.	Statistic	df	Sig.						
Tensile stress (MPa): Polypropylene-ACTM	0.194	15	0.133	**0.889**	15	**0.066**	**2.319**	**0.486**	0.125	2.050–2.588	18.497	**3.093E**−**11**
Tensile strain (%): Polypropylene-ACTM	0.168	15	0.200	**0.924**	15	**0.218**	**−15.845**	**6.731**	1.738	−19.573–12.118	**−**9.117	**2.900E**−**07**

**Table 7:** Analysis of biomechanical change in rabbits implanted with polypropylene and ACTM from 24 weeks to 48 weeks.

	Levene's test for equation of variance		*T*-test for equation of means				
	*F*	*P* value	Mean difference	SE difference	95% CI	*t*	*P* value
Tensile stress (MPa): polypropylene 48 w-24 w	0.178	0.677	**1.01**	0.321	0.348–1.664	3.131	**0.004**
Tensile stress (MPa): ACTM 48 w-24 w	0.289	0.595	**1.1**	0.244	0.597–1.595	4.501	**<0.001**
Tensile strain (%): polypropylene 48 w-24 w	0.400	0.532	0.111	1.195	−2.336–2.559	0.093	0.926
Tensile strain (%): ACTM 48 w-24 w	1.349	0.255	0.697	2.104	−3.612–5.007	0.331	0.743

## Data Availability

The data used to support the findings of this study were supplied by Minggang Wang under license and so cannot be made freely available. Requests for access to these data should be made to Minggang Wang at wmgonly@126.com.
